# Supplementary material for: The causal relationship between the human gut microbiota and pyogenic arthritis: a Mendelian randomization study
Source: Front Cell Infect Microbiol. 2024 Nov 26;14:1452480. doi: 10.3389/fcimb.2024.1452480 (PMC11629706; doi:10.3389/fcimb.2024.1452480)
Supplement: Supplementary file 1 [file DataSheet1.zip › mendelian test/ebi-a-GCST90027837.csv_forest.pdf]

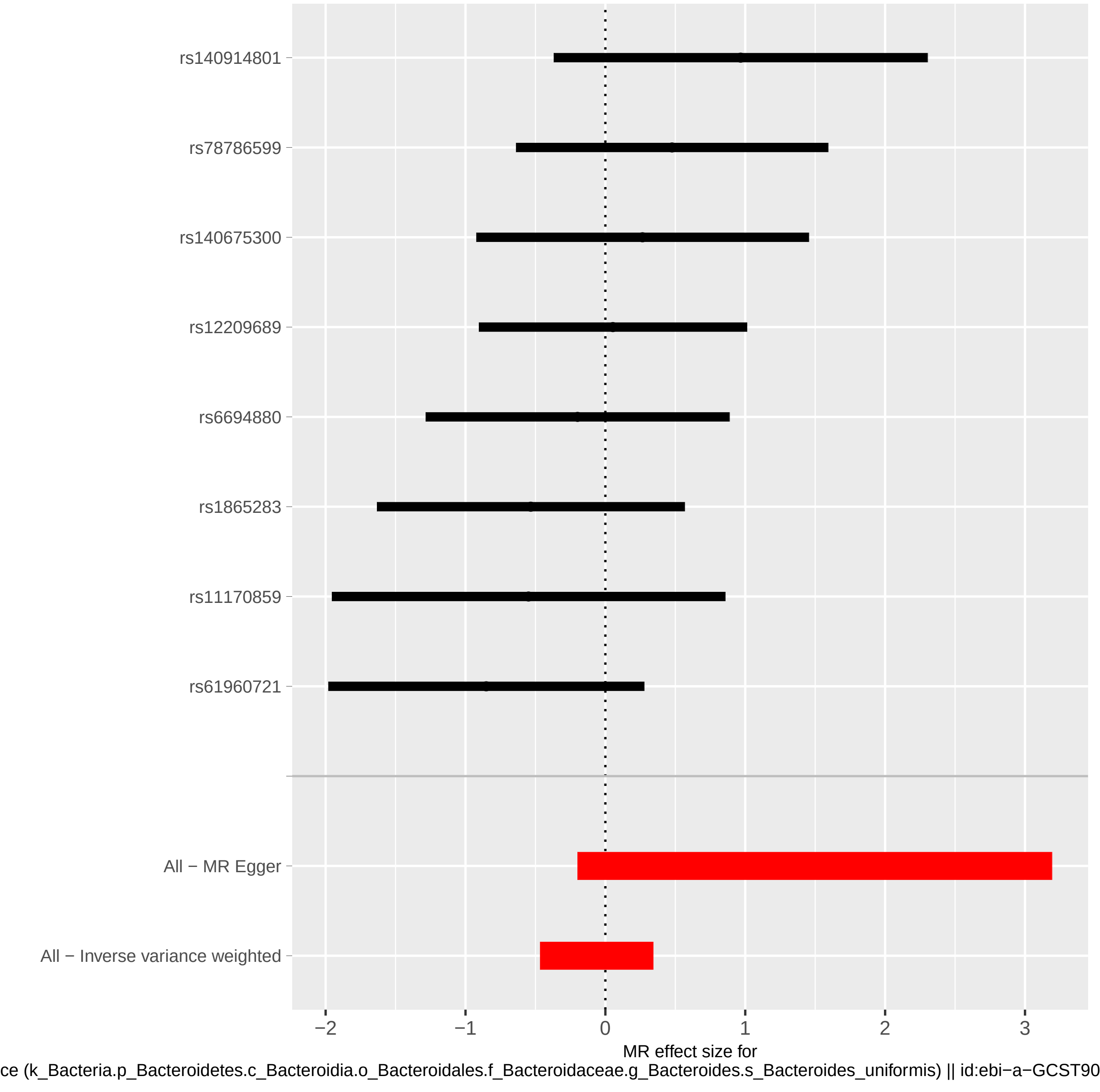
rs140914801
rs78786599
rs140675300
rs12209689
rs6694880
rs1865283
rs11170859
rs61960721
All − MR Egger
All − Inverse variance weighted
−2
−1
0
1
2
3
MR effect size for
ce (k_Bacteria.p_Bacteroidetes.c_Bacteroidia.o_Bacteroidales.f_Bacteroidaceae.g_Bacteroides.s_Bacteroides_uniformis) || id:ebi−a−GCST90
